# Haplotype structure: an overlooked key factor shaping the genomic selection response

**DOI:** 10.1093/genetics/iyag181

**Published:** 2026-07-13

**Authors:** Changyi Xiao, Viola Nolte, Christian Schlötterer

**Affiliations:** Institut für Populationsgenetik, Vetmeduni Vienna, Vienna 1210, Austria; Vienna Graduate School of Population Genetics, Vienna 1210, Austria; Institut für Populationsgenetik, Vetmeduni Vienna, Vienna 1210, Austria; Institut für Populationsgenetik, Vetmeduni Vienna, Vienna 1210, Austria

**Keywords:** experimental evolution, haplotype structure, Drosophila, polygenic adaptation, quantitative traits, Animalia

## Abstract

Genetic redundancy, a hallmark of polygenic adaptation, implies that new trait optima can be reached by frequency changes of a subset of contributing loci. As a result, the genomic response to selection is often heterogeneous among replicate populations. However, linkage disequilibrium can constrain the genomic response because linked alleles respond jointly, rather than independently. The extent to which haplotype blocks with multiple selection targets shape the genomic response during polygenic adaptation remains unclear. We tested how haplotype structure affects genomic responses to polygenic adaptation with experimental evolution. Three sets of *Drosophila simulans* lines from the same natural population (supergroups), each with 5 replicates independently adapted to the same novel temperature regime. The genomic response of replicates within the supergroup was more similar than the response of replicates from different supergroups. This pattern is not expected for freely recombining loci, but it is consistent with the central role of haplotype structure in shaping the genomic response. This effect is evident in the stark contrast between supergroups, which is greater than the differentiation caused by adaptation to 2 environments. Replicates from a hot fluctuating environment (28/18 °C) and a hot constant environment (23 °C) clustered together according to supergroup. Computer simulations supported the key role of haplotype structure on the genomic response. Our study highlights the importance of haplotype structure for polygenic selection signatures and further illustrates the stochastic nature of adaptation even when populations adapt to the same selection pressure.

## Introduction

Parallel evolution refers to the independent adaptation of populations to similar environments, resulting in alike phenotypic shifts (Jones et al. 1992; Reznick et al. 1996; Pigeon et al. 1997; Losos et al. 1998; Huey et al. 2000). However, the occurrence of parallel phenotypic evolution does not necessarily imply convergence at the genetic level, as for polygenic traits distinct genetic paths can contribute to similar phenotypic values. For example, common beans underwent 2 independent domestication events, but only 0.2% of the total number of genes show selection signals in both populations (Gaut 2015). A similar pattern was observed in 2 independent adaptations of maize to high altitude, which showed limited evidence for parallel evolution at the nucleotide level (Takuno et al. 2015). While it is conceivable that selection pressures may differ across populations due to the multidimensional nature of the natural environment (e.g. temperature and humidity), even under controlled environmental conditions, nonparallelism persists at the genomic level. *Drosophila simulans* populations originating from Portugal and Florida adapted to the same laboratory environment, but different loci contributed to the same phenotypic shift (Mallard et al. 2018; Barghi et al. 2019; Otte et al. 2021).

Nonparallel evolution at the genomic level can be driven by multiple evolutionary forces. First, genetic drift in finite populations causes stochastic changes in allele frequencies, reducing the extent of genomic parallelism across replicate populations. Moreover, the efficacy of selection is mitigated by drift, decreasing the likelihood that the same alleles consistently respond to selection across replicate populations (Kimura 1964). Second, the genetic architecture of the trait under selection influences the degree of parallelism. Unlike oligogenic traits—where a few contributing loci sweep to high frequencies—polygenic traits typically adapt through small allele frequency shifts at many loci (Pritchard et al. 2010; Pritchard and Di Rienzo 2010; Barghi et al. 2020). Genetic redundancy, a characteristic of polygenic adaptation, describes the phenomenon where more contributing loci are present in the genome than necessary for the population to reach trait optimum (Yeaman 2015; Barghi et al. 2019; Láruson et al. 2020). Consequently, only a subset of contributing loci may respond during adaptation, leading to heterogeneous genomic responses even among replicated populations adapting to the same environment. The probability of a polymorphic site responding to selection depends on its effect size and initial frequency as well as the distance to the trait optimum (Franssen et al. 2017a; Hayward and Sella 2022).

If each contributing locus responds to adaptation independently, the adaptive response becomes unpredictable, as every locus can be replaced by another one. However, linkage disequilibrium (LD) among contributing variants can influence the adaptive potential of individual SNPs, resulting in a distinct genomic response. If 2 or more loci are located on the same chromosome and contribute in the same direction to the trait, their combined fitness effect will be greater than that of each locus individually (Aeschbacher and Bürger 2014; Lotterhos et al. 2018). In this case, not only the contributing loci will rapidly increase in frequency but also neutral loci on the same chromosome. This signal of linked selection has been analyzed in a genome-wide covariance analysis of allele frequency changes from time-series data (Buffalo and Coop 2020; Lynch et al. 2024). However, if the contributing loci reside on different haplotypes and are inherited independently, they will compete against each other unless recombination places them on the same haplotype, a phenomenon known as the Hill–Robertson effect (Hill and Robertson 1966). Sachdeva and Barton (2018) showed that under the infinitesimal model, haplotype blocks with a favorable net effect can introgress into a population, even though the fitness of individuals from the residual and introgressed population did not differ in fitness.

In experimental evolution studies, a limited number of founder genotypes results in elevated LD, and modest population sizes in the laboratory lead to a further increase during the experiment (Nuzhdin and Turner 2013; Franssen et al. 2015). Despite the importance of linkage in shaping genomic response, many commonly used methods for detecting selection in natural (Vitti et al. 2013) and experimental populations (reviewed in Vlachos et al. 2019) assume independence between loci. Hence, the role of LD remains explored to a lesser extent.

**Fig. 1.**
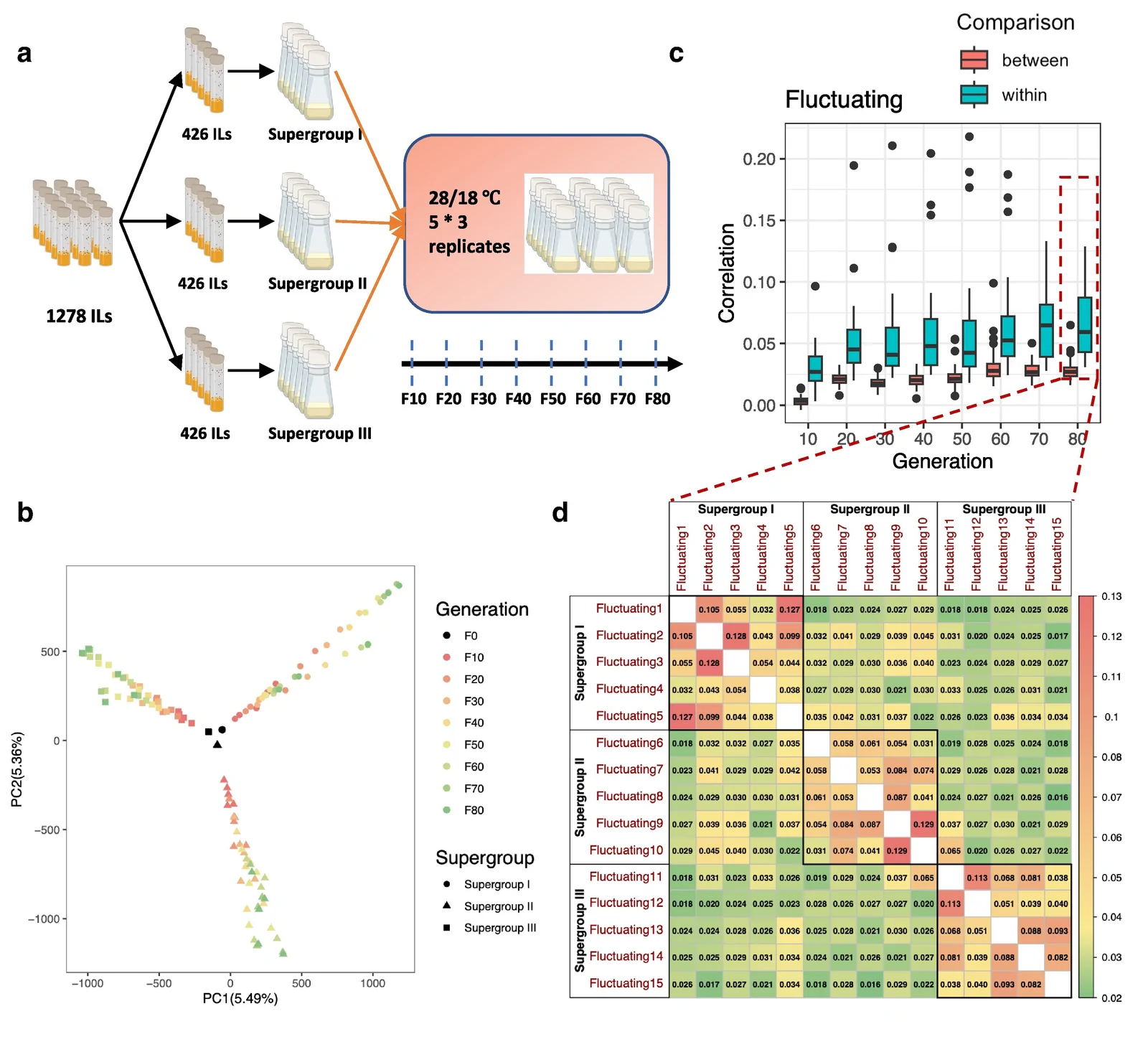
Distinct genomic responses between supergroups. a) Experimental design: A total of 1,278 isofemale lines from *D. simulans* in South Africa were evenly distributed to construct 3 supergroups of the ancestral population, and each supergroup had 5 replicates. Experimental evolution was performed at constant population size (1,250 flies) and nonoverlapping generations in a hot fluctuating environment with a 12/12 h cycle between 28 and 18 °C. b) PCA results in a distinct genomic response between supergroups. Shapes indicate supergroups, and each group contains 5 independently evolved replicates. c) Time-resolved allele frequency change correlation in the experimental evolution of populations that evolved in the hot fluctuating temperature. Within each generation, the box on the right represents within-supergroup comparisons, whereas the box on the left represents between-supergroup comparisons. d) Pearson correlation of allele frequency change from generation 0 to generation 80 between replicates that evolved to the hot fluctuating temperature. Pearson correlation coefficients are shown within each cell. Figure 1a was created in BioRender. Schlötterer, C (2026) https://BioRender.com/fgs9aqn.

To shed light on the importance of haplotype structure in shaping the genomic response, we assembled 3 supergroups, each established from 426 different isofemale lines originating from the same South African *D. simulans* population (Fig. 1a). Hence, the allele frequencies are expected to be similar across different supergroups, but they were constituted from different haplotypes. Founder populations from each of the 3 supergroups laid eggs in 2 batches, and the resulting progeny evolved under 2 laboratory temperature regimes for 80 generations: a hot temperature regime fluctuating between 18 and 28 °C and a hot constant regime at 23 °C. Our analysis focuses primarily on populations evolved under the fluctuating regimes. We observed systematic differences in the genomic responses of replicates between supergroups. We then used simulations to characterize the influence of haplotype structure on shaping the genomic responses. The best match to the empirical data with a higher similarity in genomic response among populations from the same supergroup than between supergroups was found for polygenic adaptation, but the extent of polygenicity could differ considerably. Remarkably, when the same ancestral population evolved in a hot constant environment at 23 °C, we observed that the genomic response was more distinct between supergroups than between treatments. This result suggests that haplotype structure has a greater influence than temperature regime in driving the divergent evolution in our experimental evolution.

## Materials and methods

### Experimental setup

*D. simulans* individuals were collected from South Africa in 2012 and used to establish 1,278 isofemale. These isofemale lines of the founder population were randomly assigned to create 3 supergroups, each containing 426 nonoverlapping isofemale lines. We established a replicate population, which was then exposed to the selection regime, by pooling 5 mated females from each isofemale line assigned to the corresponding supergroup; this procedure was repeated 5 times, yielding 5 replicates per supergroup and a total of 15 biological replicates across the 3 supergroups. After construction, the ancestral replicates laid eggs twice for the subsequent experimental evolution in 2 laboratory environments: a hot fluctuating temperature regime with 28 °C during the day and 18 °C at night (28/18 °C) and a hot constant temperature regime throughout the day and night (23 °C). These populations were maintained as independent replicates with a census population size of 1,250 and a 50:50 sex ratio. During experimental evolution, approximately 600 flies were sampled every 10 generations to construct libraries for Pool-Seq. The libraries were sequenced using paired-end reads [details about the sequencing platform are available at European Nucleotide Archive {ENA}]. The experiment spanned generation 0 to 80, with an average sequencing coverage of 292× for the ancestral populations and 100× for the evolved populations, allowing us to track the allele frequencies over time.

### Variant calling

Sequencing data were trimmed using in-house scripts, removing 6 bases from the 5′ end of each read. Base quality smaller than 13 was set to 0, and only reads longer than 35 bp were kept. Then the adapter sequences were removed. The trimmed reads were mapped to the *D. simulans* reference genome M252 (Palmieri et al. 2015) using bwa-mem2 (v2.2.1) (Vasimuddin et al. 2019). Then bamUtil (v1.0.14) (Jun et al. 2015) was used to clip overlaps, and duplicates were marked using MarkDuplicates (Broad Institute 2018) and removed later.

Mapped reads with a mapping quality larger than 20 and proper pairs were included using SAMtools view (v1.14). Duplicates and fragments that were shorter than the read length were removed from the data (Danecek et al. 2021). As bwa-mem sometimes assigns the maximum mapping quality of 60 to reads that are not uniquely mapped, relying on a mapping quality of 60 can lead to overconfidence in the placement of not uniquely mapped reads. To address this issue, we applied a conservative filtering based on the 2 best alignment scores in AS (alignment score) and XS (secondary alignment score) tags. We define a heuristic mapping quality based on the ratio x = AS/(AS + XS). Then the mapping probability is calculated using the function:


$$\mathrm{mapping}\;\mathrm{probability}=\frac{x^{n}}{x^{n}+{\left(1-x\right)}^{n}}$$

adapted from J. M. (2019), with *n* = 7.5. In the next step, the mapping quality is recalibrated by multiplying the re-estimated probability by the mapping quality. This approach reduces false confidence in ambiguously mapped reads while retaining informative data. Then cram files for each sample were mpileup using BCFtools with parameter “-d $MAXDEPTH –incl-flags 0 × 2 -q 10 -Q 20 -D -a AD,” max depth corresponding to 5 times the average coverage for each sample (Danecek et al. 2021). After mpileup, samples were merged using BCFtools. SNPs within repeated regions, within 5 base pairs of an indel or other variants, or with less than 50% or greater than 200% of the average coverage across samples were removed from the dataset. Frequency was determined by AD/sum(AD). Natural population samples harbor a large fraction of low-frequency SNPs. Most of them do not contribute to adaptation, but impose a major burden to the data analysis. Nevertheless, since some low-frequency SNPs can contribute to adaptation, we opted for a strategy that retains low-frequency SNPs that have reached a frequency of at least 0.15 in at least a single sample. Removing SNPs with a minor allele frequency of <0.15 in all samples (including ancestral and evolved ones) resulted in 5.3 million SNPs.

### Detecting the pattern of the genome-wide adaptive response

As 3 supergroups of the founder population originated from the same natural population, we expect a similar genomic response between the 3 supergroups that were subjected to the same laboratory environment. We checked this by visualizing the genomic change during the experimental evolution with principal component analysis (PCA) of the allele frequencies in 10-generation intervals in R (R Core Team 2023). The raw allele frequencies were arcsine square root transformed before performing the PCA to account for the different variances of rare and common SNPs. Additionally, to check the parallelism in genomic responses between independently evolved replicates, we estimated the Pearson correlation between replicates using the allele frequency change from generation 0 to generation 80. Another approach to quantify genome-wide allele frequency changes leverages the estimation of effective population sizes (*N*_e_), with small N_e_ pointing toward stronger linked selection—mediated through the joint effect of LD, selection strength, and number of contributing loci. We estimated *N*_e_ from the allele frequency changes between generation 0 and generation 80 using the poolSeq Package (Taus et al. 2017). For each replicate population, 1,000 SNPs were randomly sampled to estimate *N*_e._ This procedure was repeated 100 times (bootstrap resampling), and the median N_e_ across bootstrap replicates was recorded as the estimate specific for the corresponding replicate population (Supplementary Table 2).

### Forward simulations

We took advantage of 8,000 haploid genome sequences that were previously simulated to match a natural *Drosophila melanogaster* population with a size of 750,000 (Bastide et al. 2013). In our simulation, we denote this population sample as the full population from which supergroups were generated by random sampling haploid genomes without replacement. Forward simulations of replicate populations from 3 supergroups were performed using SLiM v4 (Haller and Messer 2023). For computational efficiency, we simulated only chromosome 2L because, for a subset of parameters, identical results were obtained when the entire genome was simulated. Simulation data were analyzed in the same way as the empirical data. The simulations are summarized in Supplementary Table 1.

#### Simulation A: LD vs LE

We used a simulation strategy similar to the experimental setup to evaluate the generality of the phenomenon observed in the empirical data. We simulated 6 different numbers of contributing loci (10, 100, 1,000, 5,000, 10,000, 20,000), 3 different total selection strengths (i.e. sum of all contributing loci; 10, 50, 100), and 2 simulations under neutrality with either a census population of 300 or 1,250 individuals. For each scenario, contributing loci were randomly sampled from the segregating sites with an equal selection coefficient and dominance coefficient of 0.5 [e.g. 100loci_s10 (sc = 0.1) indicates 100 contributing loci with a total selection strength of 10, and the corresponding selection coefficient of 0.1 per contributing locus in the simulation].

For each parameter combination, 10 independent runs (with the same number of contributing loci and selection strength, but with the contributing loci located at different positions and potentially having different initial frequencies) were simulated, resulting in a total of 3,000 simulations (20 parameter combinations × 10 runs × 3 supergroups × 5 replicates).

We simulated a sweep scenario in SLiM by introducing beneficial mutations with positive fitness effects, which are expected to increase in frequency under selection. The extent of increase depends on the selection coefficient and initial frequency. We have chosen this approach to match the early directional phase of polygenic adaptation when populations rapidly respond to a new optimum (Barghi et al. 2020; Hayward and Sella 2022). For simplicity, the simulation used 400 founder lines, a number similar to the 426 lines used to initialize the experimental evolution. Then, supergroups of the founder population in the simulations were established by sampling 400 individuals from the 8,000 haploid genomes described above. This procedure was repeated 3 times to form 3 supergroups with no overlap between the individuals from different supergroups. Given the relatively large sample size, most contributing loci are expected to be shared across all 3 supergroups. The recombination rate was taken from *D. melanogaster* (Comeron et al. 2012), and the population size was kept constant at 1,250. During the simulations, the allele frequency of all the segregating sites was recorded every 10 generations. At each sampling time point, 600 individuals were randomly sampled from the population, and allele frequencies were adjusted for Poo-Seq sampling noise by binomial sampling with a sequencing coverage of 100× per site. For the simulations under linkage equilibrium (LE), the same parameters as in the LD scenario were used, except for the recombination rate, which was set to 0.5 between all loci to test the influence of haplotype structure on genomic response. Hence, 3,000 simulations were also performed with free recombination.

**Fig. 2.**
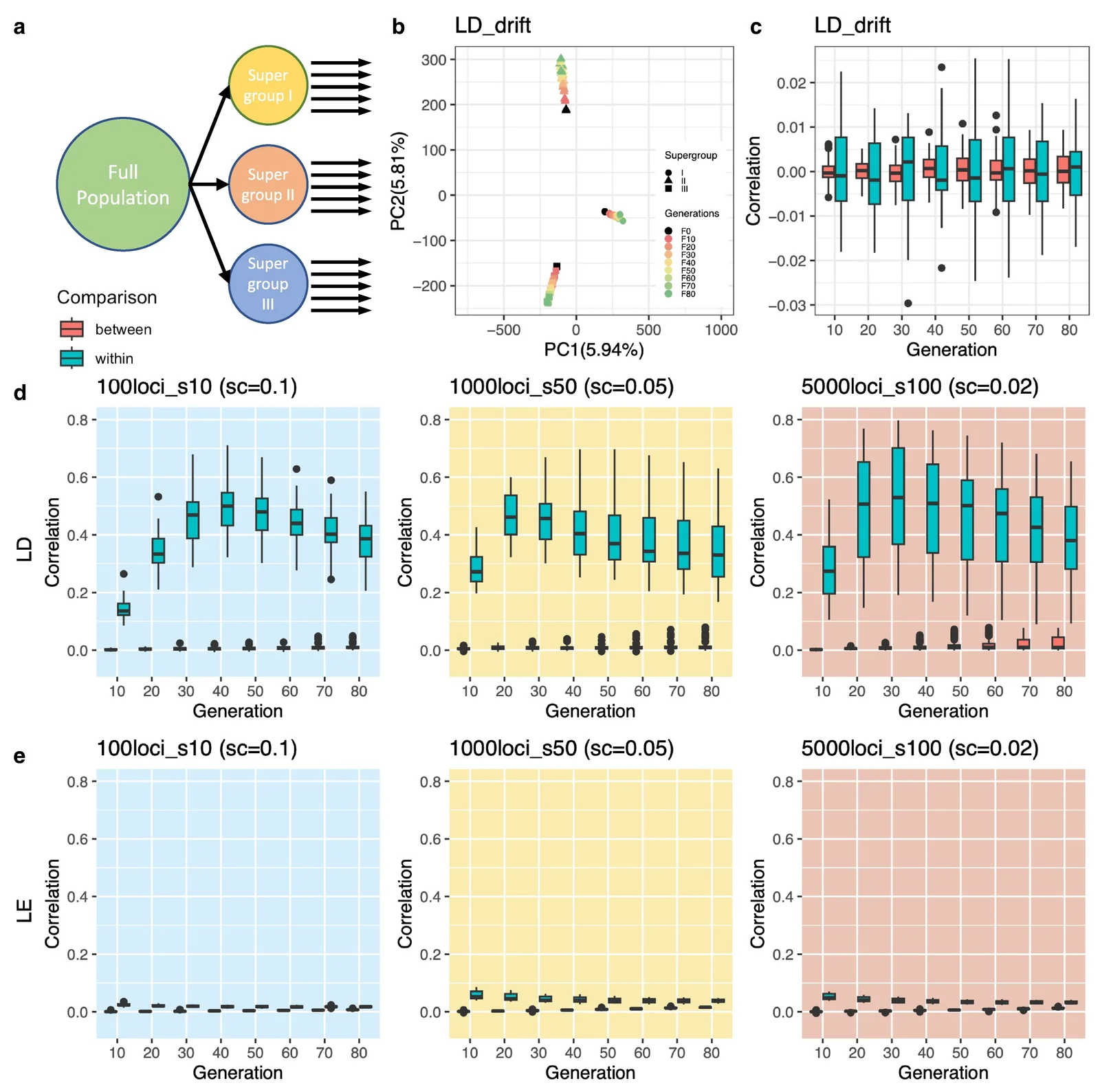
Computer simulations support the role of haplotype structure in shaping the genomic responses: LD vs LE. a) Simulation strategy: 3 supergroups of the founder population were generated by random sampling from the full population (matching a natural population). b) PCA of 1 typical neutral simulation run with LD. c) Time-resolved allele frequency change correlation in the neutral simulation with LD. d) Time-resolved allele frequency change correlation in simulations with selection under LD. e) Time-resolved allele frequency change correlation in simulations with selection under LE. The *x* axis indicates the number of generations under selection, and the *y* axis shows the Pearson correlation of allele frequency change between replicates. Within each generation, the box on the right represents within-supergroup comparisons, whereas the box on the left represents between-supergroup comparisons. The subtitle denotes the selection scenario (loci, number of selected loci; s, total additive selection strength; sc, the corresponding selection coefficient per contributing locus). Results shown correspond to the first of 10 runs, each with independently chosen selection targets; summaries across all 10 runs are provided in the Supplementary material.

Furthermore, although the differences in allele frequency correlation were much smaller under LE, they were still detectable between supergroups and within supergroups, particularly in the early generations of adaptation (Fig. 2e; Supplementary Fig. 6). This does not reflect a biological signal but reflects the logic of the SLiM software. Selection occurred before recombination in each generation (Haller and Messer 2023). Consequently, the same high-fitness haplotypes can be repeatedly inherited among replicates within a supergroup (in biological terms, this means that selection occurs before gametes are produced). This process leads to the elevated correlation in allele frequency changes observed within supergroups under LE during the early phase of adaptation.

#### Simulation B: identical starting frequency

To eliminate the possibility that the differences between supergroups are caused only by small allele frequency differences, we designed a simulation scheme that minimizes the differences in allele frequencies between supergroups. Specifically, we first randomly sampled 400 homozygous individuals. Supergroups I, II, and III were then generated by randomly shuffling alleles among these 400 individuals (rather than drawing random samples from the large population samples without replacement). This procedure ensured that all 3 supergroups had identical allele frequencies but were constituted by different haplotypes. Simulations were subsequently performed in the same way as in simulation A under both LD and LE, and a total of 6,000 simulations were performed with shuffled alleles (20 parameter combinations × 2 linkage × 10 runs × 3 supergroups × 5 replicates).

#### Simulation C: sample size

With this set of simulations, we examine whether an increase in sample size could mitigate the effect of haplotype structure in shaping the genomic responses. We test this using 1 selection regime with 100 contributing loci, each having a selection coefficient of 0.1 (a total selection strength of 10). We tested 4 different sample sizes: 400, 800, 1,200, and 2,500. For each simulation, supergroups were established by sampling individuals from the 8,000 individuals, with the number of samples corresponding to the sample size. This procedure was repeated 3 times to form 3 supergroups. Given the relatively large sample size, most contributing loci are expected to be shared across all 3 supergroups. For the simulation with a sample size of 2,500, since the census population size was set to 1,250 in the simulation, we combined every 2 homozygous samples into a single heterozygous sample. The simulation was performed in the same way in simulation A under LD, and a total of 600 simulations were performed to evaluate the influence of sample size (4 parameter combinations × 1 linkage × 10 runs × 3 supergroups × 5 replicates).

## Results

**Fig. 3.**
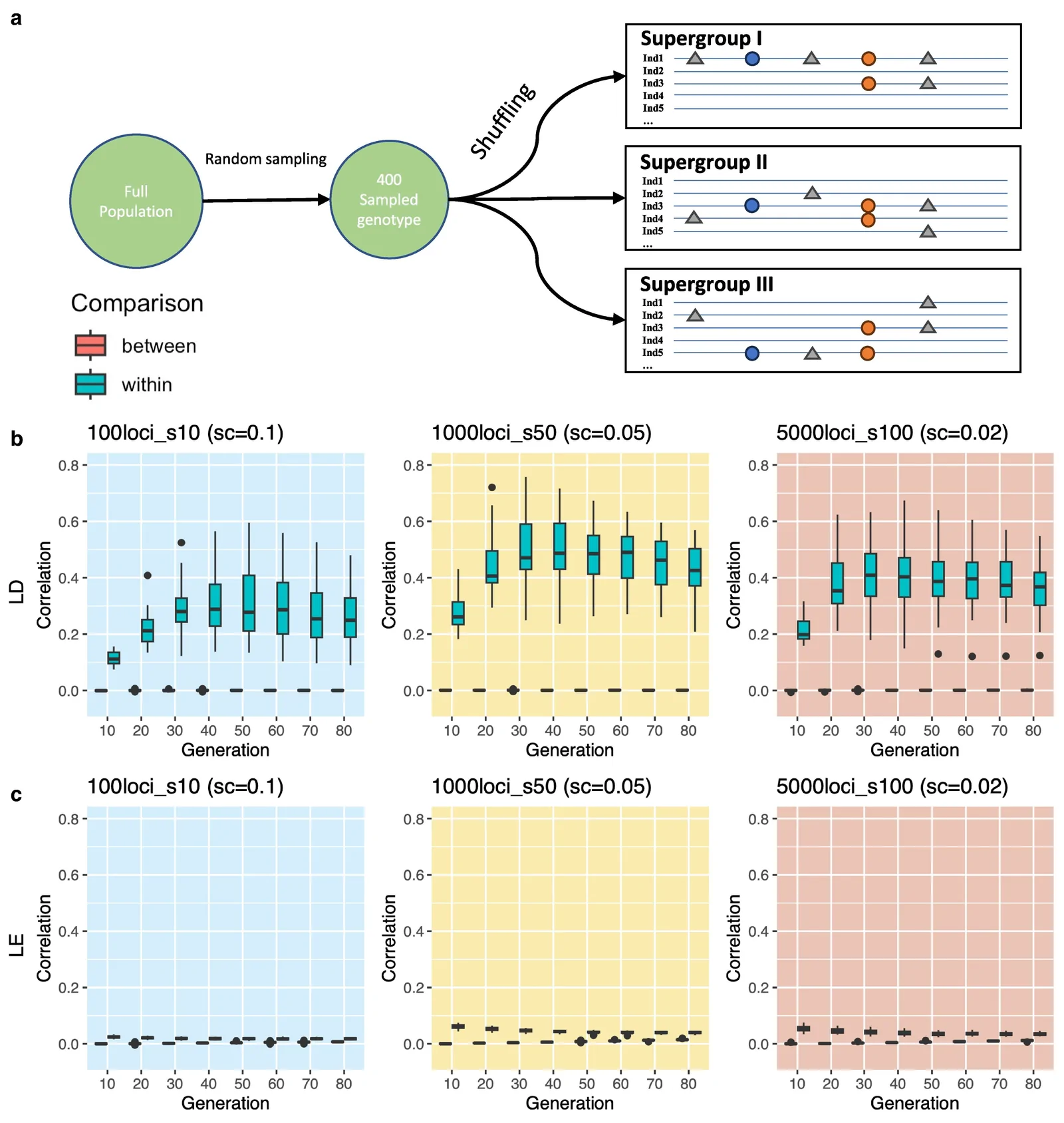
Computer simulations highlight the role of haplotype structure on the genomic response: identical starting frequency. a) Simulation strategy: 400 individuals were sampled from the full population. Supergroups I, II, and III were then generated by randomly shuffling alleles among these 400 samples. Hence, 3 supergroups have the same allele frequency, but with different haplotypes. b) Time-resolved allele frequency change correlation in simulations with selection under LD. c) Time-resolved allele frequency change correlation in simulations with selection under LE. The *x* axis indicates the number of generations under selection, and the *y* axis shows the Pearson correlation of allele frequency changes between replicates. Within each generation, the box on the right represents within-supergroup comparisons, whereas the box on the left represents between-supergroup comparisons. The subtitle denotes the selection scenario (loci, number of selected loci; s, total additive selection strength; sc, the corresponding selection coefficient per contributing locus). Results shown correspond to the first of 10 runs, each with independently selection targets; summaries across all 10 runs are provided in the Supplementary material.

**Fig. 4.**
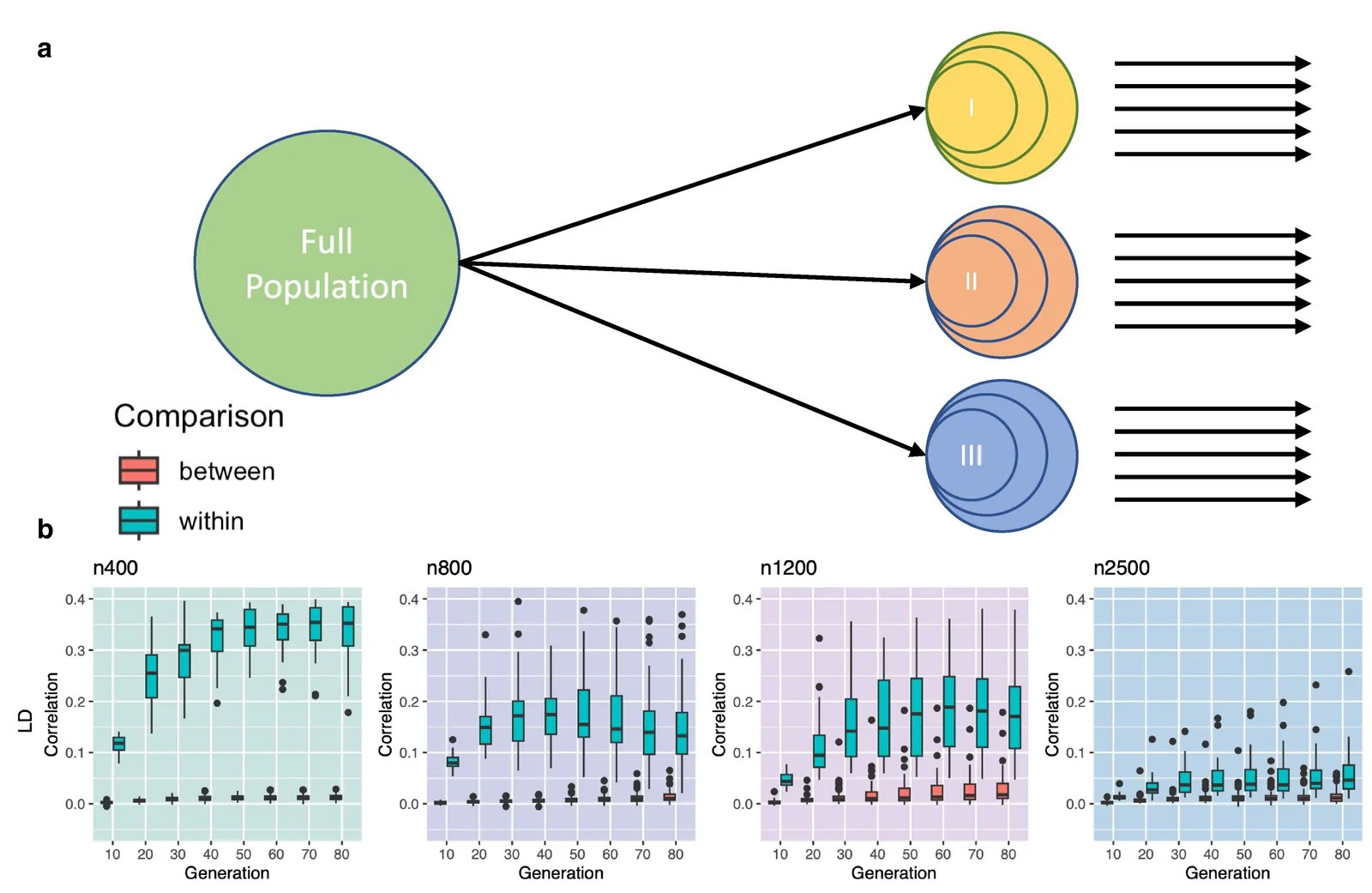
Computer simulations support the role of selection for the genomic responses: sample size. a) Simulation strategy: 3 supergroups were randomly sampled from the same full population. The size of the circles reflects the number of haplotypes sampled from the full population (400, 800, 1,200, and 2,500). b) Time-resolved allele frequency change correlation in simulations with selection under LD. The *x* axis indicates the number of generations under selection, and the *y* axis shows the Pearson correlation of allele frequency changes between replicates. Within each generation, the box on the right represents within-supergroup comparisons, whereas the box on the left represents between-supergroup comparisons. Simulations were performed for populations with selection regime 100loci_s10 (sc = 0.1) under LD. The subtitle denotes the sample size used to reconstruct the ancestral population. Results shown correspond to the first of 10 runs, each with independently chosen selection targets; summaries across all 10 runs are provided in the Supplementary material.

**Fig. 5.**
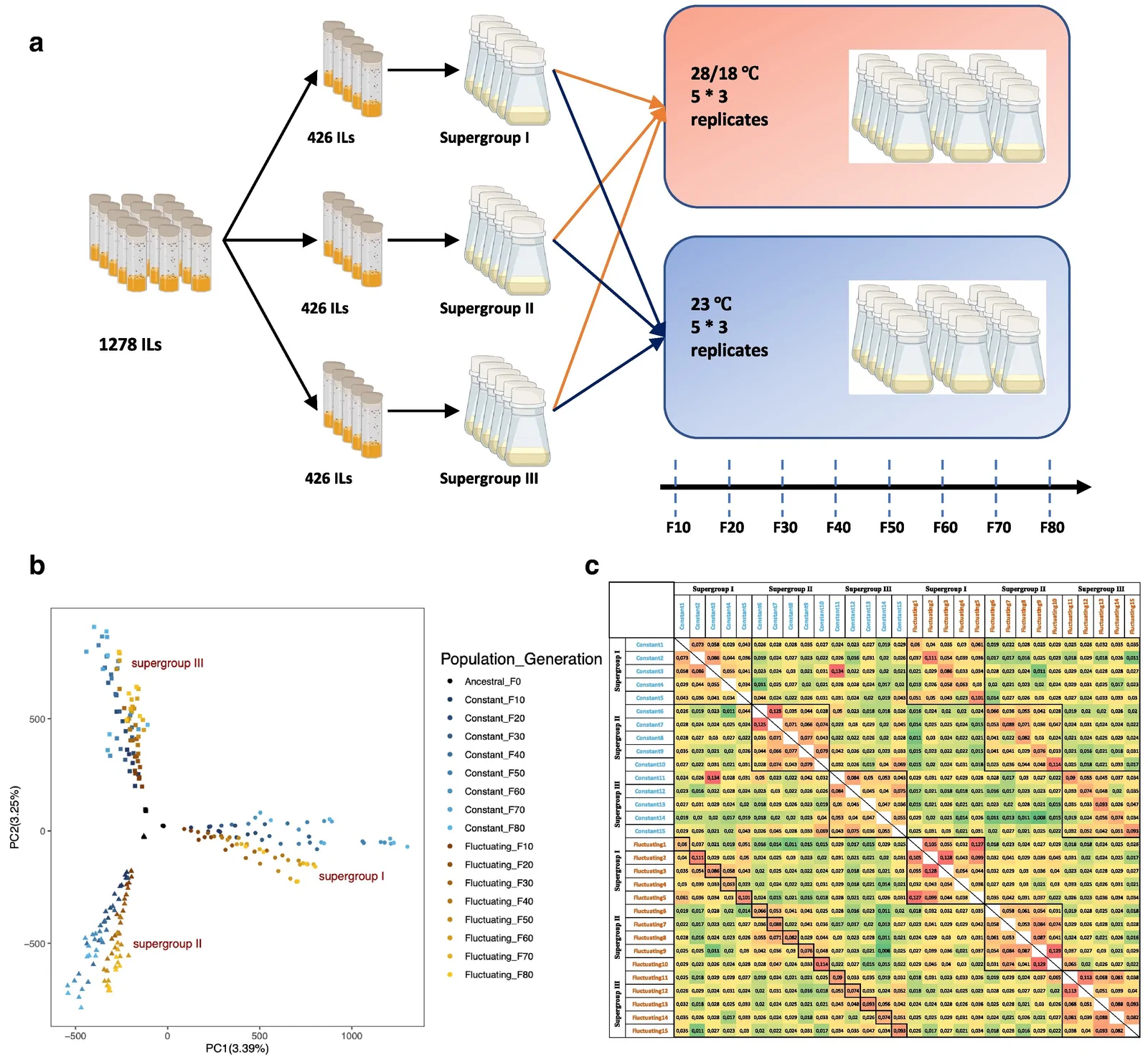
Distinct genomic responses are more pronounced between supergroups than between treatments. a) Experimental design: A total of 1,278 isofemale lines from *D. simulans* in South Africa were evenly distributed to construct 3 supergroups of the ancestral population, and each supergroup had 5 replicates. Experimental evolution was performed at constant population size (1,250 flies) and nonoverlapping generations in a hot fluctuating environment with a 12/12 h cycle between 28 and 18 °C and in a hot constant environment at 23 °C. b) PCA results in a more pronounced genomic divergence between supergroups than between selection regimes. Colors indicate the selection regime, shapes indicate supergroups, and each group contains 5 independently evolved replicates. c) Pearson correlation of allele frequency change from generation 0 to generation 80 between replicates that evolved to the hot fluctuating temperature and hot constant temperature regime. Pearson correlation coefficients are shown within each cell. Figure 5a was created in BioRender. Schlötterer, C (2026) https://BioRender.com/tzxtido.

We performed experimental evolution to compare the adaptive responses of 3 independent population samples (supergroups) that originated from the same natural *D. simulans* population collected in South Africa. A total of 1,278 isofemale lines were distributed into 3 supergroups, and 5 replicate populations were established from each supergroup by pooling the corresponding isofemale lines. Founder replicates were generated in 2 egg-laying batches, and their progeny were evolved for 80 generations in 2 laboratory environments: a hot fluctuating temperature regime cycling between 18 and 28 °C (Fig. 1a) and a hot constant temperature regime at 23 °C (Fig. 5a). We mainly focused on populations adapting to the fluctuating environment (Figs. 1–4), but we also compared the genomic response between the 2 environments (Fig. 5).

### Systematic differences in genomic responses between supergroups

Out of 5.3 million high-confidence SNPs, 5.1 million SNPs (95.43%) are shared across all 3 supergroups (Supplementary Fig. 1). With such a large number of SNPs segregating in all 3 supergroups, we hypothesize that they share most of the contributing loci and have the potential to respond to selection in all 3 supergroups. We tested our hypothesis with a PCA of the allele frequency time series in 10-generation intervals. Surprisingly, despite the similar allele frequencies of the 3 ancestral supergroups, the genomic response of evolved populations diverged between supergroups (Fig. 1b). The Pearson correlation of allele frequency change showed the consistent pattern of replicates from the same supergroup having a higher correlation than those between supergroups (Fig. 1c and d). This suggests that replicates within the same supergroup have a higher similarity in the genomic response to selection.

### Computer simulations supporting the key role of haplotype structure

To evaluate the generality of the distinct responses of supergroups during experimental evolution, we performed forward simulations resembling the experimental setup. The founder population was generated by a random sample of 400 homozygous individuals from 8,000 simulated homozygous individuals (full population), and this procedure was repeated 3 times to form 3 supergroups from the founder population (Fig. 2a). We used PCA and the Pearson correlation coefficient of allele frequency change between the evolved population and ancestral population to measure the similarity in genomic response between replicates and supergroups. Our simulations focused on selection strength and the number of contributing loci because we considered them the most informative parameters. In the absence of reliable estimates, we included rather extreme values—between 10 and 20,000 contributing loci and a total selection strength ranging from 10 to 100. Although our goal was not to infer the number of contributing loci or the selection strength in our data, we nevertheless estimated the *N*_e_ based on the observed allele frequency changes as an approximate indicator of the parameter combination yielding the highest compatibility with the empirical results. In the main document, we only show results of parameters that provide a similar *N*_e_ estimate as the empirical data (Fig. 2c and d). The *N*_e_ estimates for the complete set of runs are given in the supplement (Supplementary Fig. 2). The results for the complete set of parameter combinations are shown in the supplement (Supplementary Figs. 3 and 4).

Overall, the simulations mirrored our empirical results, with the first and second PC separating the supergroups, while replicates within a supergroup were not distinguishable (Supplementary Fig. 3). Notably, this included neutral simulations suggesting that haplotype structure and drift alone could separate the 3 supergroups (Fig. 2b). Five simulated scenarios produced a different pattern, with PC1 mainly separating ancestral and evolved populations. A closer inspection showed that increased selection strength and fewer contributing loci were driving this pattern of a parallel selection response (Supplementary Fig. 3).

As another readout of the genome-wide response, we measured the allele frequency change correlation within and between supergroups. Most simulations matched the empirical result of a higher correlation within than between supergroups. This pattern was observed across populations with different numbers of contributing loci and selection coefficients (Fig. 2d; Supplementary Fig. 4), but not for neutral simulations. Overall, the pattern of higher similarity of populations that share the same haplotype structure (same supergroup) is a general pattern regardless of the genetic architecture underlying selected traits. Neutrality and strong selection on a small number of loci (i.e. oligogenic selection) can, however, be excluded by the joint analysis of PCA and allele frequency correlations.

Typical experimental evolution studies use moderate population sizes (around 1,000 individuals) and not very many generations (less than 100); therefore, large blocks of the original haplotype structure are maintained in the population due to a limited number of recombination events (Franssen et al. 2015, 2017b). Hence, we re-run the simulations increasing the recombination rate to 0.5 to break up the haplotype structure and observed that the differences in allele frequency change within the supergroup and between supergroups were negligible (Fig. 2e; Supplementary Fig. 6). Although the PCA of simulations under LE separates the supergroups, this mainly reflects the differences between ancestral supergroups—selection had a much smaller impact on the PCA results under LD (Supplementary Figs. 3 and 5). In summary, our simulations showed that the joint effect of haplotype structure and selection is shaping the genomic response.

As a result of the sampling procedure, the supergroups are constituted by different sets of isofemale lines with distinct haplotype combinations. Thus, it is possible that not all contributing loci are shared among the supergroups, especially since low-frequency SNPs will be private to a single supergroup. This is reflected in the empirical data, where most of the SNPs were segregating in all 3 supergroups, but about 10% of the SNPs are polymorphic only in 1 or 2 supergroups (Supplementary Fig. 1). Hence, such SNPs can only respond to selection if they segregate in the respective supergroups. Furthermore, even for SNPs shared across all 3 supergroups, the allele frequencies may differ slightly. To rule out that the distinct selection response among supergroups in the empirical data is primarily caused by the genetic differences between the supergroups at the beginning of the experiment, we performed an extra set of simulations. We used a common set of contributing loci that were shuffled among haplotypes in the 3 supergroups (Fig. 3a). This procedure maintained distinct linkage structures for the same set of contributing loci. The simulations also resulted in a higher correlation among replicates from the same supergroup than across supergroups, and without linkage, the difference disappeared. These results validated the key role of haplotype structure in shaping the genomic responses (Fig. 3b and c; Supplementary Figs. 7 to 10). Moreover, when checking the PCA results of the neutral simulations under simulation B (identical starting allele frequencies), the replicates from different supergroups were intermingled, suggesting that the “trio-structure” of the PCA observed in simulation A (LD vs LE) is the result of systematic differences in allele frequencies between supergroups in the ancestral population (Fig. 2b; Supplementary Figs. 3 and 7).

### Increasing sample size could mitigate the effect of haplotype structure

Selection targets are typically identified by sampling individuals randomly from natural populations. Although random samples from a natural population closely match the allele frequency of the natural population, each random sample contains a different set of haplotypes. Our results showed that the distinct haplotype structure of random samples from the same natural population determines sample-specific selection responses. Therefore, the selection signatures detected may differ between experiments because distinct haplotypes were sampled from the same natural population. Because heterogeneity in selection outcome triggered by sampling is undesirable, we reasoned that a larger sample size could reduce differences between supergroups by capturing greater haplotype diversity. This in turn can minimize the effects of linked selection. We tested this hypothesis using simulations with varying sample sizes (Fig. 4a). As expected, the supergroups are most distinct on a PCA with a small sample size, and this separation becomes increasingly weaker with increasing sample size (Supplementary Fig. 11). In concordance with these observations, the differences in allele frequency change correlation between supergroups and within supergroups are smaller when the sample size increases (Fig. 4b; Supplementary Fig. 12). However, these differences persisted even when 2,500 haplotypes were sampled—the maximum possible in a diploid population with a census population size of 1,250.

### The genomic response between supergroups is more pronounced than between treatments

Previously, we showed that the genomic selection signature clearly differs between replicate populations from the same founder isofemale lines that were subjected to 2 different temperature regimes, either constant 23 °C or fluctuating between 18 and 28 °C (Fig. 5a). We were interested to relate these environmentally triggered selection differences to haplotype-driven signals. We compared the 3 supergroups after 80 generations of evolution in these 2 temperature regimes. Surprisingly, the largest differences shown in PC1 and PC2 were between supergroups (Fig. 5b and c) rather than between treatments. This suggests that the genomic response was more influenced by the differences between supergroups than by the differences in the treatments tested. Nevertheless, despite the smaller differences between treatments within each supergroup compared to the differences between supergroups, observable differences between treatments still exist, suggesting that treatment-specific stressors also shape the genomic response.

## Discussion

Our experimental evolution study revealed striking differences in the genomic selection signatures of 3 independent samples obtained from the same natural population. This difference was much larger than the heterogeneity among replicates within a sample, so we concluded that it is best explained by the combined effect of haplotype structure and selection. The importance of haplotype structure was further demonstrated by the genomic response of replicates exposed to 2 different temperature selection regimes, which was dominated by the supergroup rather than the selection regime.

Although we did not directly measure phenotypic changes or fitness, several lines of evidence indicate that we observed a response to selection in our data. First, simulations under neutrality suggest that the *N*_e_ should approximate the census population size (Supplementary Fig. 2). However, our estimates of *N*_e_ from the experimental evolution populations are substantially lower than the census size (Supplementary Table 2). This observation indicates that the allele frequency changes in the experimental populations were larger than expected from drift alone, providing genomic evidence for selection. Second, the PCA of the empirical data separated the replicate populations from the 2 temperature treatments (Fig. 5b). Under pure drift, replicate populations from both temperature treatments would be intermingled. Third, previous work in our laboratory with populations evolving under the same laboratory conditions demonstrated phenotypic adaptation (Mallard et al. 2018; Barghi et al. 2019; Christodoulaki et al. 2022; Lai and Schlötterer 2022; Hsu et al. 2024). This suggests that similar selection pressures also caused fitness increases in the current study. Taken together, these observations make it highly likely that selection drove the response of the fly populations to the laboratory environment.

The computer simulations confirmed the importance of haplotype structure for the selection response. However, neither PCA nor the correlation analysis alone was sufficient to align simulated data with empirical observations. This could be seen in the case of neutral evolution where drift differentiated the 3 supergroups in the PCA, similar to the empirical data; however, the correlation analysis for neutral data could not distinguish between within-supergroup and between-supergroup replicates (Fig. 2,b and c). Similarly, strong selection on the contributing loci produced the expected pattern, with higher correlation within supergroup than between supergroups (Fig. 2d; Supplementary Fig. 4). However, the PCA indicated a highly parallel response on PC1 (Supplementary Fig. 3). These discordant patterns are important for interpreting our empirical data because we can exclude pure drift and strong selection on a small number of loci. Including *N*_e_ estimates from the observed allele frequency changes suggests that a moderate level of polygenicity (i.e. 100 loci with a selection strength of 10) could explain the observed empirical data.

Moreover, we caution that this analysis is too simplistic to infer the full adaptive architecture, as the simulations assumed equal selection coefficients for all contributing loci. While this facilitated the comparison of populations with different numbers of contributing loci and kept our simulations computationally feasible, it does not fully reflect biological reality. Nevertheless, since our study focuses on the role of haplotype structure in shaping genomic response, this simplification is unlikely to affect our conclusions.

Our study shows that the selection response is contingent on the identity of the haplotypes used to set up the founder population. This implies that experiments initiated from distinct founder genotypes most likely generate different selection signatures. While we observed this effect already for genotypes sampled from the same natural population, the effect will be even more pronounced for experiments using founder populations derived from distinct natural populations. Additionally, many experimental evolution studies start from a much smaller number of founder genotypes than in this study (Orozco-Terwengel et al. 2012; Mallard et al. 2018; Barghi et al. 2019; Rudman et al. 2022; Bitter et al. 2024). These reduced number of founder genotypes further limits the similarity between experimental outcomes and the expected response of natural populations—even when identical selection pressures are applied. Furthermore, recombination among a moderate number of founder genotypes prior to the selection experiment (Phillips et al. 2021) does not solve this issue as it creates new haplotypes, which are not present in natural populations. Therefore, we recommend including a large number of founder genotypes to obtain a selection response, which is more representative of the natural population. Overall, our study highlights an important but often underappreciated factor contributing to nonparallel genomic evolution across studies employing different base populations. We do not regard this dependence on the founder haplotypes as a major problem, as long as it is clear that genetic redundancy implies that alternative genetic solutions are expected when using other founder genotypes.

Our results are also important for interpreting (non)parallel selection responses in natural populations. Evolutionary biologists often rely on parallel genomic selection signatures to identify selection targets (Turner et al. 2011; Orozco-Terwengel et al. 2012; Tobler et al. 2014; Nouhaud et al. 2016; Barghi et al. 2017). While this is undoubtedly an effective strategy for excluding false positives, our results suggest that this dependence on parallel selection signatures is overly conservative. Different founder genotypes are likely to result in distinct selection signatures. Therefore, focusing on parallel selection signatures will result in a large number of false negatives, which is particularly true when founder populations are based on a small number of founder genotypes.

Chromosomal inversions result in an interesting form of haplotype structure. Many studies have demonstrated that inversions are frequently selected, and this selection signal is highly parallel, even across continents (Reinhardt et al. 2014; Fang et al. 2020). We propose that independent samples of chromosomal inversions from different populations will exhibit distinct dynamics than those described here. Since inversions are usually functionally divergent, sometimes even originate from different species (Reeve et al. 2024; Stankowski et al. 2024), we hypothesize that the fitness differences between inverted and noninverted alleles are much greater than those between recombining chromosomes. Consequently, inversions are expected to act as strongly selected super loci. In our simulations, stronger selection typically leads to more parallel selection signatures. Therefore, we expect inverted regions to exhibit more parallel responses, with initial frequency differences playing only a minor role.

In summary, our results—based on both experimental evolution and forward simulations—demonstrate that the haplotype structure of the founder population plays a crucial role in shaping the genomic response to selection. Remarkably, this effect can even exceed the influence of the selective treatment itself. Our findings highlight the importance of haplotype structure in shaping polygenic selection signatures and further underscore the stochastic nature of adaptation, even when populations evolve under identical selection pressures.

## Supplementary Material

iyag181_Supplementary_Data

## Data Availability

Raw sequencing data from the experimental evolution study have been deposited in the ENA under project accession PRJEB98374. The code used for the analysis of the empirical data and simulation is available on GitHub at https://github.com/cyxiao94/haplotype_stucture_data_code.git. We did not store the simulation data but provide the random seed number for full reproducibility of the simulations. Supplemental material available at *GENETICS* online.
