# Supplementary material for: Haplotype structure: an overlooked key factor shaping the genomic selection response
Source: Genetics. 2026 Jul 13;234(1):iyag181. doi: 10.1093/genetics/iyag181 (PMC13535250; doi:10.1093/genetics/iyag181)
Supplement: Supplementary file 1 [file iyag181_supplementary_data.docx]

**Supplementary Information**

**Table S1. Summary of the simulations**

|  | Description | Rational | parameters | Number of parameters | Number of simulations | Figures |
| --- | --- | --- | --- | --- | --- | --- |
| Simulation scheme A | founder populations were generated by random sampling | resembling the experimental setup to evaluate the generality of the haplotype structure | Number of contributing loci | 6 | (6*3+2)*2*10*15=6000 | Fig. 2  Fig. S3  Fig. S4  Fig. S5  Fig. S6 |
|  |  |  | Selection strength | 3 |  |  |
|  |  |  | Extra simulations* | 2 |  |  |
|  |  |  | Linkage | 2 |  |  |
|  |  |  | Simulation runs | 10 |  |  |
|  |  |  | Supergroup | 3 |  |  |
|  |  |  | Replicates | 5 |  |  |
| Simulation scheme B | founder populations were generated by shuffling alleles | minimize the allele frequency differences among supergroup to better focus on the role of haplotype structure | number of contributing loci | 6 | (6*3+2)*2*10*15=6000 | Fig. 3  Fig. S7  Fig. S8  Fig. S9  Fig. S10 |
|  |  |  | selection strength | 3 |  |  |
|  |  |  | Extra simulations* | 2 |  |  |
|  |  |  | Linkage | 2 |  |  |
|  |  |  | Simulation runs | 10 |  |  |
|  |  |  | Supergroup | 3 |  |  |
|  |  |  | Replicates | 5 |  |  |
| Simulation scheme C | founder populations were randomly sampled with different sample size | test the influence of sample sizeon the haplotype structure | Sample size | 4 | 4*1*10*15=600 | Fig. 4  Fig. S11  Fig. S12 |
|  |  |  | Linkage | 1 |  |  |
|  |  |  | Simulation runs | 10 |  |  |
|  |  |  | Supergroup | 3 |  |  |
|  |  |  | Replicates | 5 |  |  |

*extra simulations indicate two neutral simulations with population size of 300 and 1250 respectively

**Table S2. Estimated effective population size of the experimental populations**

| Supergroup | Replicates | Treatment | | | |
| --- | --- | --- | --- | --- | --- |
|  |  | **28/18 ℃** | | **23 ℃** | |
|  |  | **autosome** | **X** | **autosome** | **X** |
| Supergroup I | **r1** | 257 | 310 | 208 | 158 |
|  | **r2** | 323 | 247 | 190 | 199 |
|  | **r3** | 375 | 338 | 251 | 272 |
|  | **r4** | 329 | 273 | 216 | 267 |
|  | **r5** | 269 | 273 | 215 | 172 |
| Supergroup II | **r1** | 259 | 274 | 212 | 209 |
|  | **r2** | 303 | 272 | 249 | 276 |
|  | **r3** | 326 | 300 | 265 | 256 |
|  | **r4** | 310 | 301 | 227 | 182 |
|  | **r5** | 321 | 293 | 249 | 266 |
| Supergroup III | **r1** | 299 | 275 | 286 | 261 |
|  | **r2** | 356 | 310 | 208 | 199 |
|  | **r3** | 288 | 229 | 210 | 187 |
|  | **r4** | 272 | 203 | 201 | 212 |
|  | **r5** | 320 | 331 | 289 | 242 |

**Fig. S1. Characterization of the ancestral population.** A) Venn diagram shows the number of SNPs that are segregating in each supergroup and shared across supergroups at generation 0. Three supergroups shared most of the segregating site B) Boxplot showing pairwise Fst comparing replicates within and between supergroups at generation 0.

**Fig. S2. Estimated effective population size (N_e_) using allele frequency data from simulated data based on generation 0 and generation 80.** Colored boxes indicate estimated N_e_ from simulation scheme A with different selection regimes under LD. The white box indicates N_e_ from the neutral simulation. The Red dashed line represents the median N_e_ estimated from the empirical data under 28/18°C. The X-axis indicates different selection regimes in the simulation, the y-axis shows the estimated N_e_, and panels correspond to different simulation runs. The lower panel shows the same analysis, but for all 10 simulation runs which are based on independently chosen selection targets.

**Fig. S3. PCA of allele frequency time series simulated under a broad range of selection strength and number of contributing loci (simulation scheme A: random sampling under LD).** Colors indicate generations that evolved in the selection regime, the shape indicates supergroups, and each group contains five independently evolved replicates. Subtitle denoted the selection scenario (loci: number of selected loci; s: total selection strength; sc: the corresponding selection coefficient per contributing locus). Results shown are from simulation Run1, and results from other runs are available at the provided GitHub link.

**Fig. S4. Correlation of allele frequencies for replicates within and between supergroups simulated under a broad range of selection strengths and number of contributing loci (simulation scheme A: random sampling under LD).** Correlations were estimated from all segregating sites using allele frequency changes between generations 0 and 80. The X-axis indicates the number of generations under selection, and the y-axis shows the median Pearson correlation of allele frequency changes between replicates. The median allele frequency change correlation was calculated as follows: 1) pairwise correlations were calculated within each simulation run; 2) the median correlation from each run was stored; 3)the boxplots summarize these median correlations across runs. Blue boxes represent within-supergroup comparisons, and red boxes represent between-supergroup comparisons. The background color denotes the selection scenario (loci: number of selected loci; s: total selection strength; sc: the corresponding selection coefficient per contributing locus).

**Fig. S5. PCA of allele frequency time series simulated under a broad range of selection strength and number of contributing loci (simulation scheme A: random sampling under LE).** Colors indicate generations that evolved in the selection regime, the shape indicates supergroups, and each group contains five independently evolved replicates. Subtitle denoted the selection scenario (loci: number of selected loci; s: total selection strength; sc: the corresponding selection coefficient per contributing locus). Results shown are from simulation Run1, and results from other runs are available at the provided GitHub link.

**Fig. S6. Correlation of allele frequencies calculated for replicates within and between supergroups. Results are shown for simulations under a broad range of selection strengths and number of contributing loci (simulation scheme A: random sampling under LE).** Correlations were estimated from all segregating sites using allele frequency changes between generations 0 and 80. The X-axis indicates the number of generations under selection, and the y-axis shows the median Pearson correlation of allele frequency changes between replicates. For the median allele frequency change correlation, pairwise correlations were first calculated within each simulation run, and the median correlation from each run was stored; the boxplots summarize these median correlations across runs. Blue boxes represent within-supergroup comparisons, and red boxes represent between-supergroup comparisons. The background color denotes the selection scenario (loci: number of selected loci; s: total selection strength; sc: the corresponding selection coefficient per contributing locus).

**Fig. S7. PCA of allele frequency time series simulated under a broad range of selection strength and number of contributing loci (simulation scheme B: identical starting frequency under LD).** Colors indicate generations that evolved in the selection regime, the shape indicates supergroups, and each group contains five independently evolved replicates. Subtitle denoted the selection scenario (loci: number of selected loci; s: total selection strength; sc: the corresponding selection coefficient per contributing locus). Results shown are from simulation Run1, and results from other runs are available at the provided GitHub link.

**Fig. S8. Correlation of allele frequencies calculated for replicates within and between supergroups. Results are shown for simulations under a broad range of selection strengths and number of contributing loci (simulation scheme B: shuffling genotype under LD).** Correlations were estimated from all segregating sites using allele frequency changes between generations 0 and 80. The X-axis indicates the number of generations under selection, and the y-axis shows the median Pearson correlation of allele frequency change between replicates. For the median allele frequency change correlation, pairwise correlations were first calculated within each simulation run, and the median correlation from each run was stored; the boxplots summarize these median correlations across runs. Blue boxes represent within-supergroup comparisons, and red boxes represent between-supergroup comparisons. The background color denotes the selection scenario (loci: number of selected loci; s: total selection strength; sc: the corresponding selection coefficient per contributing locus).

**Fig. S9. PCA of allele frequency time series simulated under a broad range of selection strength and number of contributing loci (simulation scheme B: identical starting frequency under LE).** Colors indicate generations that evolved in the selection regime, the shape indicates supergroups, and each group contains five independently evolved replicates. Subtitle denoted the selection scenario (loci: number of selected loci; s: total selection strength; sc: the corresponding selection coefficient per contributing locus). Results shown are from simulation Run1, and results from other runs are available at the provided GitHub link.

**Fig. S10. Correlation of allele frequencies calculated for replicates within and between supergroups. Results are shown for simulations under a broad range of selection strengths and number of contributing loci (simulation scheme B: identical starting frequency under LE).** Correlations were estimated from all segregating sites using allele frequency changes between generations 0 and 80. The X-axis indicates the number of generations under selection, and the y-axis shows the median Pearson correlation of allele frequency change between replicates. For the median allele frequency change correlation, pairwise correlations were first calculated within each simulation run, and the median correlation from each run was stored; the boxplots summarize these median correlations across runs. Blue boxes represent within-supergroup comparisons, and red boxes represent between-supergroup comparisons. The background color denotes the selection scenario (loci: number of selected loci; s: total selection strength; sc: the corresponding selection coefficient per contributing locus).

**Fig. S11. PCA of allele frequency time series simulated under different number of sample size (simulation scheme C: sample size under LD).** Colors indicate generations that evolved in the selection regime, the shape indicates supergroups, and each group contains five independently evolved replicates. Subtitle denoted the selection scenario (loci: number of selected loci; s: total selection strength; sc: the corresponding selection coefficient per contributing locus). Results shown are from simulation Run1, and results from other runs are available at the provided GitHub link.

**Fig. S12. Correlation of allele frequencies calculated for replicates within and between supergroups. Results are shown for simulations under different sample sizes (simulation scheme C: sample size under LD).** Correlations were estimated from all segregating sites using allele frequency changes between generations 0 and 80. The X-axis indicates the number of generations under selection, and the y-axis shows the median Pearson correlation of allele frequency change between replicates. For the median allele frequency change correlation, pairwise correlations were first calculated within each simulation run, and the median correlation from each run was stored; the boxplots summarize these median correlations across runs. Blue boxes represent within-supergroup comparisons, and red boxes represent between-supergroup comparisons. The background color denotes the selection scenario (n: number of sampled haplotypes in the founder population).
